# First person – Laura Kuil and Nynke Oosterhof

**DOI:** 10.1242/dmm.039420

**Published:** 2019-03-08

## Abstract

First Person is a series of interviews with the first authors of a selection of papers published in Disease Models & Mechanisms (DMM), helping early-career researchers promote themselves alongside their papers. Laura Kuil and Nynke Oosterhof are co-first authors on ‘Reverse genetic screen reveals that Il34 facilitates yolk sac macrophage distribution and seeding of the brain’, published in DMM. Laura is a PhD student in the lab of Dr Tjakko van Ham at Erasmus University Medical Center, Rotterdam, The Netherlands, investigating the genetic regulation of microglia/macrophage development and their function. Nynke is a postdoc in the lab of Dr Judith Paridaen at University Medical Center Groningen, Groningen, The Netherlands, investigating cortical neurogenesis.


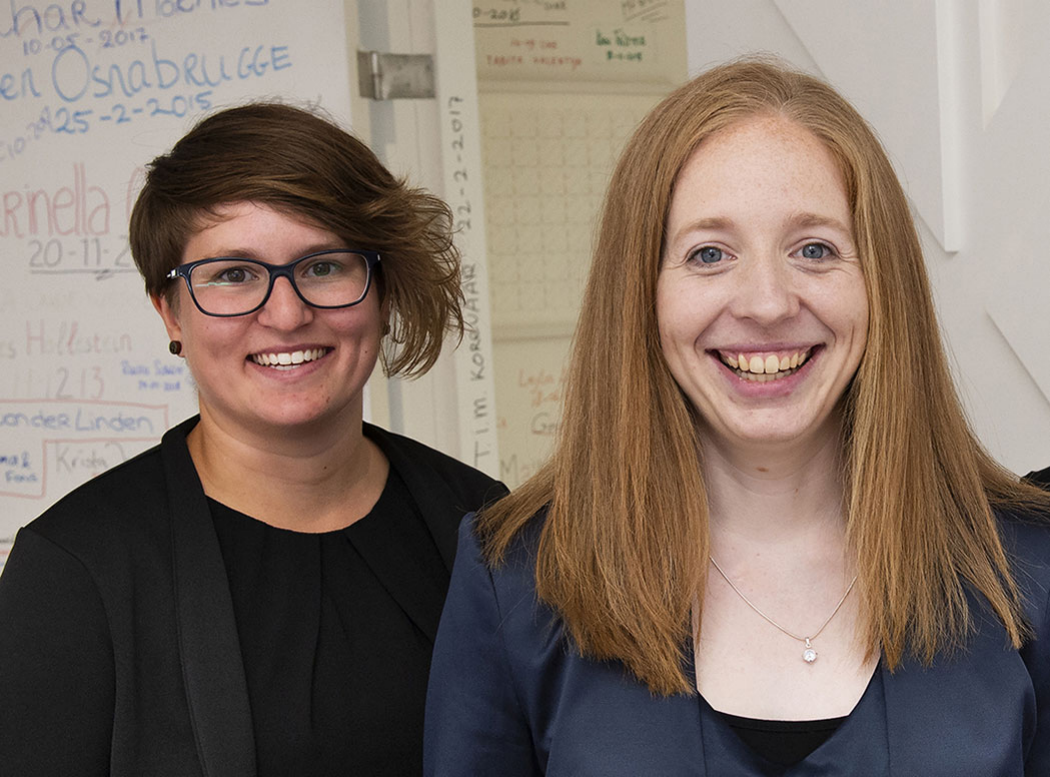


**Laura Kuil (left) and Nynke Oosterhof (right)**

**How would you explain the main findings of your paper to non-scientific family and friends?**

LK: Microglia are the immune cells of the brain and are involved in many brain diseases (e.g. Alzheimer's disease). In the healthy brain, microglia are quite busy cells that remodel connections between cells, clean all types of waste material and support the formation of myelin. Microglia develop from progenitor cells that arise on the yolk sac macrophages (YSMs), which migrate towards the brain during embryonic development. How their development is regulated remains largely undiscovered. Therefore, we developed a strategy to switch off genes one by one, and identify genes that affect the early development of microglia in a living organism, the zebrafish. We found that a signaling molecule, called Il34, facilitates the migration of YSMs towards the brain and other tissues. These findings bring us one step closer to understanding what regulates the journey of microglia progenitor cells to the brain during development, which is important for the therapeutic potential of microglia and, for example, the replacement of defective microglia in the diseased brain.

NO: I would start to say that the brain consists of different types of cells, including microglia, which are the cells that are important for the development and protection of our brains. In fact, mistakes in genes that, in the brain, appear to be specifically expressed in microglia have been identified as causes for severe brain diseases as well as brain malformations. During early embryonic development, microglia precursors develop on the yolk sac, after which they travel to the brain to become microglia. However, the exact genes and mechanisms involved in the development of microglia are still not completely known and understood. In this study, we used zebrafish to perform a small-scale genetic screen to identify genes that are important for the development of microglia. In this screen, we tested 20 genes and identified a gene called *il34* as a regulator of microglia development. Additional experiments revealed that this gene, which encodes a signaling molecule, is necessary for microglia precursor cells to find their way to the brain.

**What are the potential implications of these results for your field of research?**

LK and NO: In this study, we showed that we were able to successfully identify genes that are important for microglia development with our reverse genetic screening method in zebrafish. We tested only 20 genes; however, it is possible to increase the throughput of this screening method. This would allow for a relatively rapid way to identify new regulators of microglia development. This would be particularly interesting in the context of brain diseases with potential/certain microglia involvement. In addition, microglia are very difficult to culture, as their phenotype highly depends on their environment. Because of this, they lose their brain-specific properties as soon as they are taken into a cell culture environment. Insight into their developmental regulation could help with the development of better culturing protocols, as well as assist in, for example, the development of induced pluripotent stem cell-derived microglia and their delivery to the brain. Our small-scale screen identified Il34 as an important regulator of microglia development. The role of Il34 in early microglia development was, based on previous studies, controversial. Our findings show direct evidence for a role of Il34 in early microglia development.

“What surprised me is the high efficiency of gene targeting we could reach in injected zebrafish by using CRISPR/Cas9 complex injections.”

**What are the main advantages and drawbacks of the model system you have used as it relates to the disease you are investigating?**

NO and LK: The main reason for using zebrafish in this study is that with CRISPR/Cas9-based genome editing it is possible to get a very high throughput in a relatively short amount of time compared to mammalian model organisms. In zebrafish, the first microglia have appeared and started to colonize the brain within 3 days after fertilization. Also, the embryos are transparent and can easily take up chemicals including dyes from the water, which allowed us to label microglia and determine their numbers several days after fertilization in live embryos. Additionally, they have a high fecundity and often lay >100 fertilized eggs per breeding pair. Finally, mutagenesis by CRISPR/Cas9-based genome editing works so efficiently that mutant phenotypes can be created directly in injected animals. A drawback of this model system could be that, evolutionarily speaking, zebrafish are not as closely related to humans as mammals. However, given that many tissues, cell types and genes are conserved between zebrafish and mammals, there is a high chance that genes that are important for microglia development in the zebrafish also play a role in mammalian microglia development.

**Figure:**
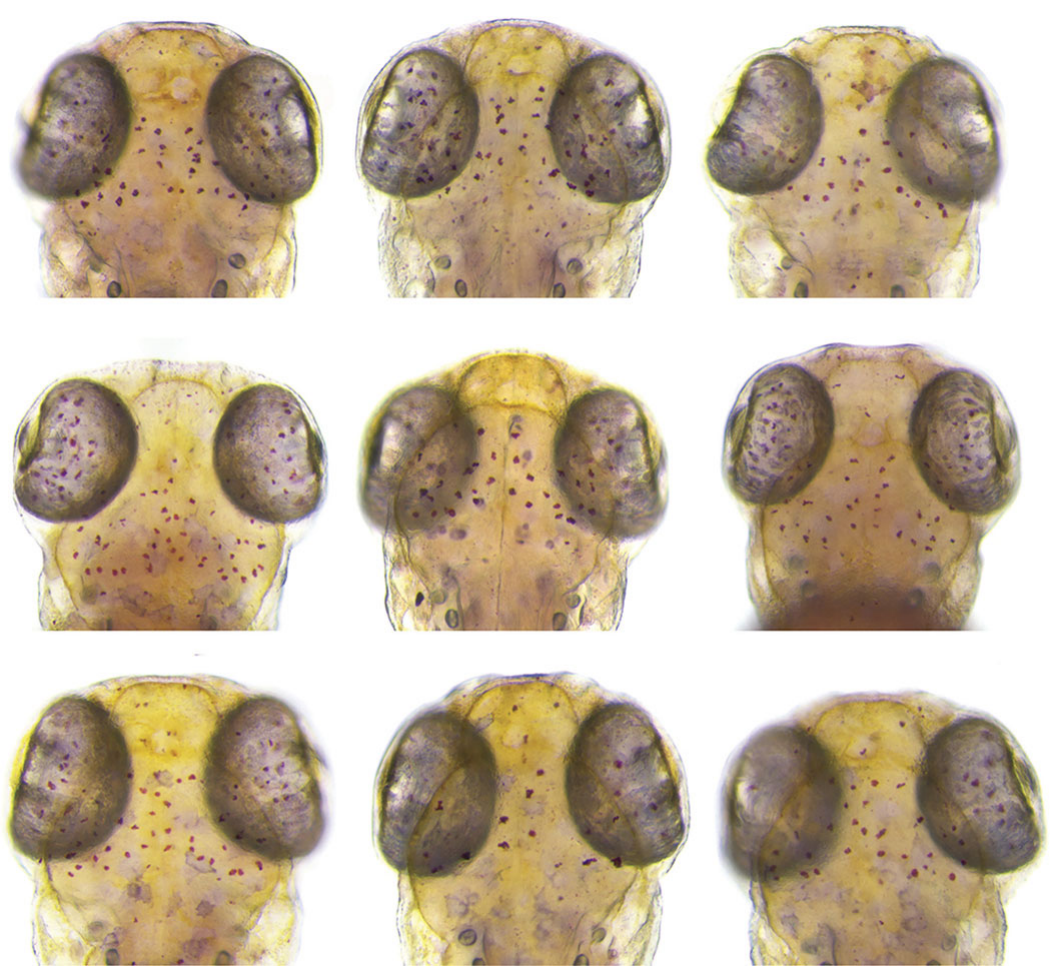
**Images of zebrafish larvae stained with Neutral Red, which labels the microglia used in the reverse genetic screen.**

**What has surprised you the most while conducting your research?**

NO: At the moment, I can't think of anything that really surprised me during my research. One of the things I really liked though was that for the development of our SpotNGlia software tool we worked together with people with completely different backgrounds, such as physics. I thought it was really interesting to see how we looked at our research question from a completely different point of view. I think this experience was very valuable and really helped me to look at problems from different perspectives.

LK: What surprised me was the high efficiency of gene targeting we could reach in injected zebrafish by using CRISPR/Cas9 complex injections. This enabled us to directly analyze ‘crispants’ within a workweek, which nicely speeds up the process of identifying genes affecting – in this case – microglia, but this is applicable to countless cell types and disease-related questions.

**Describe what you think is the most significant challenge impacting your research at this time and how will this be addressed over the next 10 years?**

LK: With respect to availability of, for example, antibodies and conditional knockout approaches, I think the zebrafish field (but also that of other genetic models such as *C. elegans* and *Drosophila*) is still catching up with the mouse field. Within the next 10 years, I think this will dramatically change, especially with the success of applying genome editing using CRISPR/Cas9 and other or modified versions, to generate patient-specific mutations and reporter lines in zebrafish.

NO: I think that the focus will be more and more on things that happen at the single-cell level. Even though, with *in vivo* imaging studies (for which the zebrafish is an ideal model organism), it is possible to study cell behavior at the single-cell level, it would be very useful if this could be directly related to cellular heterogeneity identified by single-cell omics techniques. Some techniques have already been developed that allow for real-time visualization of transcription and I think these kinds of imaging techniques will be more widely used and further developed in the coming years.

“[…] it is always a good idea for a PhD student to have a back-up plan, in terms of side projects, in case your ‘main’ project does not go as planned.”

**What changes do you think could improve the professional lives of early-career scientists?**

NO: I think this is a very difficult question. I could say things like more funding and opportunities, but I am not really sure whether this is all that is important. I also think that there are things that we as early-career scientists can do ourselves. For example, I think that, in the coming years, bioinformatics will become more and more important in biological research and that, as a biologist, having some bioinformatic skills will become a necessity. Therefore, I think that trying to get that kind of knowledge, for example, through courses or by talking to people with the necessary expertise will be important.

LK: In my opinion, increased possibilities for early-career scientists to give oral presentations at conferences and more funding possibilities for ‘high risk-high gain’ projects for young scientists would improve their professional lives. From my own experience I can say that it is always a good idea for a PhD student to have a back-up plan, in terms of side projects, in case your ‘main’ project does not go as planned.

**What's next for you?**

LK: I will soon defend my PhD thesis and after that start a new challenge, perhaps a nice postdoc position working with zebrafish.

NO: After defending my thesis last year, I started as a postdoc on a project in which I will try to gain more insight into the mechanisms involved in cortical neurogenesis.

**What where the most fun activities you did with your labmates?**

LK: One of our current students is very good at a form of rock climbing called ‘bouldering’, so we went bouldering with all our group members and she taught us how to do it – it was so much fun!

NO and LK: Escape rooms!

## References

[DMM039420C1] Kuil L. E., Oosterhof N., Geurts S. N., van der Linde H. C., Meijering E. and van Ham T. J. (2019). Reverse genetic screen reveals that Il34 facilitates yolk sac macrophage distribution and seeding of the brain. *Dis. Model. Mech.* 12, dmm037762 10.1242/dmm.037762 30765415 PMC6451432

